# Decoding Microbial Responses to Ammonia Shock Loads in Biogas Reactors through Metagenomics and Metatranscriptomics

**DOI:** 10.1021/acs.est.3c07840

**Published:** 2023-12-19

**Authors:** Maria Gaspari, Gabriele Ghiotto, Victor Borin Centurion, Thomas Kotsopoulos, Davide Santinello, Stefano Campanaro, Laura Treu, Panagiotis G. Kougias

**Affiliations:** ‡ Soil and Water Resources Institute, Hellenic Agricultural Organisation Dimitra, Thermi, Thessaloniki 57001, Greece; § Department of Hydraulics, Soil Science and Agricultural Engineering, School of Agriculture, Aristotle University of Thessaloniki, Thessaloniki 54124, Greece; ⊥ Department of Biology, University of Padova, Padova 35121, Italy

**Keywords:** anaerobic digestion, ammonia shock, microbiome, metagenomics, metatranscriptomics, osmotic balance, methanogenesis

## Abstract

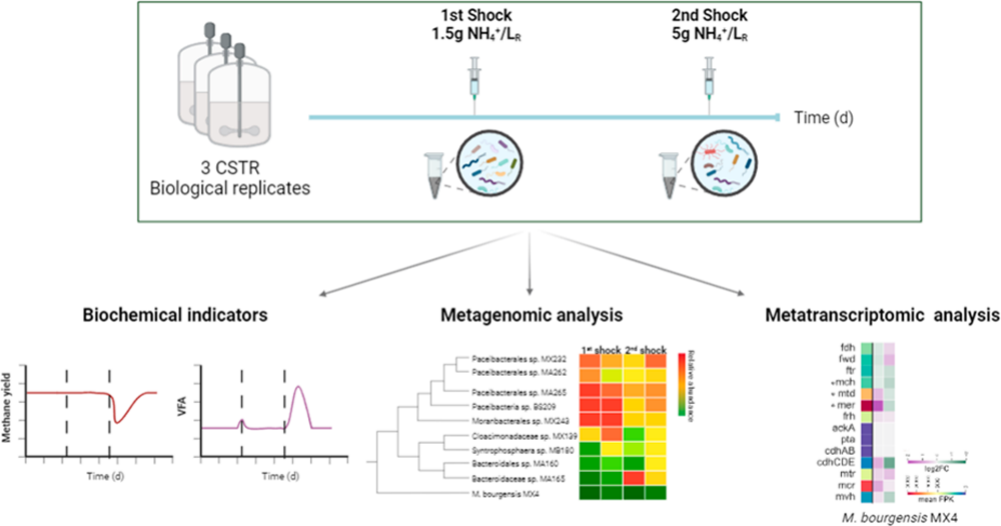

The presence of elevated ammonia levels is widely recognized as a significant contributor to process inhibition in biogas production, posing a common challenge for biogas plant operators. The present study employed a combination of biochemical, genome-centric metagenomic and metatranscriptomic data to investigate the response of the biogas microbiome to two shock loads induced by single pulses of elevated ammonia concentrations (i.e., 1.5 g NH_4_^+^/L_R_ and 5 g NH_4_^+^/L_R_). The analysis revealed a microbial community of high complexity consisting of 364 Metagenome Assembled Genomes (MAGs). The hydrogenotrophic pathway was the primary route for methane production during the entire experiment, confirming its efficiency even at high ammonia concentrations. Additionally, metatranscriptomic analysis uncovered a metabolic shift in the methanogens *Methanothrix* sp. MA6 and *Methanosarcina flavescens* MX5, which switched their metabolism from the acetoclastic to the CO_2_ reduction route during the second shock. Furthermore, multiple genes associated with mechanisms for maintaining osmotic balance in the cell were upregulated, emphasizing the critical role of osmoprotection in the rapid response to the presence of ammonia. Finally, this study offers insights into the transcriptional response of an anaerobic digestion community, specifically focusing on the mechanisms involved in recovering from ammonia-induced stress.

## Abstract 2

Ammonia is a common stressor in biogas reactors. However, the transcriptomic response of the microbial community to sudden ammonia shocks remains poorly understood. This study decodes the recovery mechanisms of ammonia-induced stress in biogas systems at the molecular level.

## 1 Introduction

Biogas production through anaerobic digestion (AD) is gaining increasing attention as it combines the reduction of environmental impacts from agricultural and industrial procedures while also providing a source of renewable energy.^1^ However, as a biological process, AD is highly sensitive to exogenous factors, and inhibitory substances in the influent feedstock can lead to operational problems.

One of the most common causes for AD process inhibition and suboptimal utilization of the biogas potential of the feedstocks is the presence of high ammonia concentration in the digesters.^2^ Ammoniacal nitrogen is a ubiquitous compound found in various organic wastes, including animal manure, food waste, and sewage sludge, which are used as an influent feedstock in biogas plants.^3^ In AD, nitrogen plays a vital role in microbial metabolism, as it is essential for bacterial growth; yet excessive levels may result in severe process imbalances.^4^ In an aqueous environment, ammoniacal nitrogen can be found in two main forms: as total ammonium ions (NH_4_^+^) and as free ammonia (NH_3_), with the latter being considered the most toxic form.^2^ It is generally accepted that among the different functional groups of microorganisms involved in the AD process, methanogenic archaea are primarily impacted by high levels of ammonia.^5^ Specifically, the inhibition of acetoclastic methanogens, which are more sensitive to the ammonia presence compared to hydrogenotrophs, regulates the acetate conversion to methane through syntrophic acetate oxidation (SAO).^6^ Typically, a broad range of total ammonia concentrations between 1000 and 3000 mg/L can inhibit the activity of AD systems depending on pH levels, while concentrations exceeding 3000 mg/L have been proved toxic to the operation regardless of the pH range.^7^ The progressive ammonia acclimatization approach has been found to be an effective mitigation strategy, demonstrating the tolerance of methanogenic consortia and allowing them to survive at high ammonia levels.^8^

The inhibitory effects of ammonia on microbial activity are complex and multifaceted, involving both direct and indirect mechanisms. Free ammonia (FAN) flows through the cell membrane by passive diffusion into the intracellular space and is converted to NH_4_^+^ by protonation.^3^ The after-effects are proton imbalance, intracellular pH change, or potassium deficiency.^9^ The presence of free ammonia can also cause severe osmotic stress or inactivate enzymes involved in organic matter digestion and methanogenesis.^10^ Additionally, a side effect of increased ammonia levels is the accumulation of VFA and H_2_.^11^ The VFA produced during the acidogenesis step cannot flow into the methanogenesis process, resulting in their accumulation.^12^

The present study aimed to unveil the early response of the AD microbiome to the introduction of ammonia shock loads in biogas reactors, employing a combined biochemical, genome-centric metagenomic, and metatranscriptomic approach. The investigation was conducted using triplicate continuous stirred tank reactors (CSTRs) exposed to ammonia shock loads induced by single pulses at two different concentrations. This comprehensive study was carried out over a period of 224 days. Steady-state conditions were established prior to subjecting the reactors to the initial shock load, which occurred on day 122. Subsequently, the reactors resumed their standard operational mode and received a second shock load on day 194, after achieving stability. Metagenomics identified the most relevant microbial taxa and their putative metabolic functions, while metatranscriptomics sheds light on microbial activity. The integrated data set from the analyses provided an enhanced understanding of the biogas microbial community during ammonia stress.

## 2 Materials and Methods

### 2.1 Inoculum and Feedstock Origin

The inoculum used was obtained from LAGADAS SA, a full-scale biogas plant located in Lagadas, Greece, treating agro-industrial and animal wastes under mesophilic conditions. The feedstock used was cattle manure, which was also provided by the same biogas plant. The manure was sieved by using a separating net with a mesh size of 2 mm to prevent clogging of the pumping tubes. Subsequently, both inoculum and cattle manure underwent a comprehensive characterization, encompassing assessments of Total Solids (TS), Volatile Solids (VS), TAN levels (NH_4_^+^-N concentration) following APHA standard methods,^13^ pH measurements, and Volatile Fatty Acids (VFA) determination based on the protocols outlined in the following section. An overview of the main biochemical characteristics of inoculum and cattle manure is provided in Table S1 (Supporting Information). Following characterization, the sieved feedstock was stored at −20 °C.

### 2.2 Experimental Setup and Process Monitoring

The experiment was conducted for a total period of 224 days in three lab-scale CSTRs (representing independent biological replicates), denoted as AR1, AR2, and AR3, operated under mesophilic conditions (37 ± 1 °C). The effective working volume of the reactors was 1.5L, and the Hydraulic Retention Time (HRT) was set to 25 days. The Organic Loading Rate of the reactors was set to 2 gVS/L_R_/d. The influent substrate was transferred to the reactors twice a day using peristaltic pumps (323 series, Watson Marlow, England). To ensure homogeneity in the overall system, reactors were continuously mixed using magnetic stirrers, while the influent bottles were mixed for 30 min before feeding.

Genomic samples were collected from the liquid fraction of each reactor before and after two shock loads. Once the startup period had been completed and stable operating conditions were established (day 106), DNA and RNA were extracted from the three reactors on day 121 of operation. Then, on day 122 a single shock load was induced in the reactors by adding 1.5 g of NH_4_–N/L (time point “I”). DNA and RNA were then extracted again 14 h after the ammonia injection. The reactors were monitored until they recovered from the shock load. Once stable operation was reestablished, a second shock load of ammonia of 5 g NH_4_–N/L was introduced into the reactors on day 194 of operation (time point “II”). The DNA and RNA sampling strategy remained consistent, including the collection of samples 1 day before and 14 h after the shock. The sampling within 14 h was determined based on a previous strategy in AD reactors ensuring that the transcriptional response due to the induced perturbation would be measurable.^14^ The time interval for the second extraction was strategically selected to capture variations in transcriptional activity while avoiding major changes in the microbial composition.

Furthermore, essential process indicators of biogas production and composition, as well as intermediate metabolites (VFA) were monitored throughout the entire experimental period. The measurement of the daily biogas production was carried out using a water replacement gas meter, and methane content was determined using a gas chromatograph (GC-2014, Shimadzu, Japan) equipped with a thermal conductivity detector (TCD) based on the protocol described by Rossi et al. (2022).^15^ The pH was determined after sampling with a pH meter (HI2020–02, HANNA instruments, USA). VFA measurements were performed in a gas chromatograph (GC-2010, Shimadzu, Japan) equipped with a Flame Ionization Detector (FID).^15^ The compounds were separated by a capillary column (ZB-FFAP, 30m, 0.53 mm I.D. × 1 μm film thickness). In addition, TAN levels were measured throughout the operation, specifically before and after the introduction of the two ammonia shock loads, to monitor the FAN concentration (Table S2) that was calculated based on the eq 1:
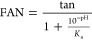
1where TAN is the total ammonium nitrogen and *K*_a_ is the dissociation constant with the value of 1.29 × 10^–9^ at mesophilic conditions.

### 2.3 Nucleic acid Extraction and Sequencing

DNA was extracted using the RNeasy PowerSoil DNA Elution Kit (QIAGEN, Hilden, Germany), while total RNA was extracted using the RNeasy PowerSoil Total RNA Kit (QIAGEN, Hilden, Germany), following the recommended protocols. To remove rRNA (rRNA) from the RNA samples, a QIAseq FastSelect Kit (QIAGEN, Hilden, Germany) was employed. The DNA and RNA libraries were prepared using the Nextera DNA Library Preparation Kit (QIAGEN, Hilden, Germany) protocol and sequenced in the NGS sequencing facility of the Biology Department (Padua, Italy) using the Illumina NextSeq 500 platform (Illumina Inc., San Diego, CA). The sequence data can be accessed in the Sequence Read Archive (SRA) database under accession number PRJNA983118.

### 2.4 Metagenomics and Metatranscriptomics

Metagenomic analyses were carried out as previously described.^12^ Briefly, the reads were coassembled using MEGAHIT v1.2.9.^16^ The depth file for the binning analyses was generated with Bowtie2 v2.4.5.^17^ Binning was performed using a combination of tools, namely MetaBAT2 v2.12.1,^18^ MaxBin v2.2.6,^19^ and Concoct v1.1.0–0.^20^ MAGs quality was evaluated with checkM v1.1.3^21^ and dereplicated using DAS Tools v1.1.2–0.^22^ Only MAGs with medium and high-quality according to MIMAGS specification^23^ were further processed. MAGs taxonomy was assigned with GTDB-Tk v 2.1.0.^24^ Finally, Prodigal v2.6.3^25^ and eggnog-mapper v2.1.9^26^ were used to predict the open reading frames and annotate them, while relative abundance (RA), read count and RPKM of the MAGs were estimated using CoverM v0.6.1.^27^ MAGs phylogenesis was assessed using PhyloPhlAn 3.0.51^28^ and the tree was visualized using iTOL v6.5.8.^29^

RNA-seq reads were processed for quality,^30^ aligned on the assembly using Bowtie2,^17^ and gene fragment counts were computed with HTSeq 2.0.2^31^ in stranded mode. DESeq2^32^ 3.14 was employed for transcriptome analysis, treating each MAG separately. Differential expression analysis (“shock + timepoint” design) was conducted using a Wald test, with a false discovery rate threshold of 0.05 and a Log2 Fold Change (log_2_FC) threshold of 1. Normalization was done using DESeq2 Estimate Size Factor, followed by Fragments Per Kilobase (FPK) calculation for gene expression quantification and visualization, allowing for direct comparison of the expression level between different genes.

### 2.5 Statistics, Metabolic Reconstruction, and Visualization

A principal component analysis (PCA) biplot was generated with Vegan v2.6–4^33^ package on R v4.2.1 using the median values of DESeq2^32^ normalization step applied on the read count computed with coverM v0.6.1,^27^ and physicochemical concentration with “rda” and “envfit” functions. Physicochemical data were normalized using the “decostand” function with the method “standardize”, which scaled the community data to zero mean and unit variance. The best subset of environmental variables with maximum correlation with microbial community (bioenv)^34^ was applied to see the Spearman correlation rank of physicochemical concentration utilizing Bray–Curtis dissimilarities with Euclidean metrics. Lastly, the magnitude and significance of the log_2_FC in the RA of the selected MAGs compared to the overall microbial community were assessed using the Mann–Whitney U test.

The reference database consulted for enzyme class, orthology and metabolic pathways was the Kyoto Encyclopedia of Genes and Genomes (KEGG).^35^ The general functional trait profile was determined with MicrobeAnnotator v2.0.4,^36^ while some specific features, including osmoprotectant synthesis, potassium uptake and energy conservation related-genes, were revised manually to check the presence/absence. Figure S1 reports results relevant to biogas production and ammonia overload (Supporting Information).

## 3 Results and Discussion

### 3.1 Biochemical Monitoring of the Reactors

**Figure 1:**
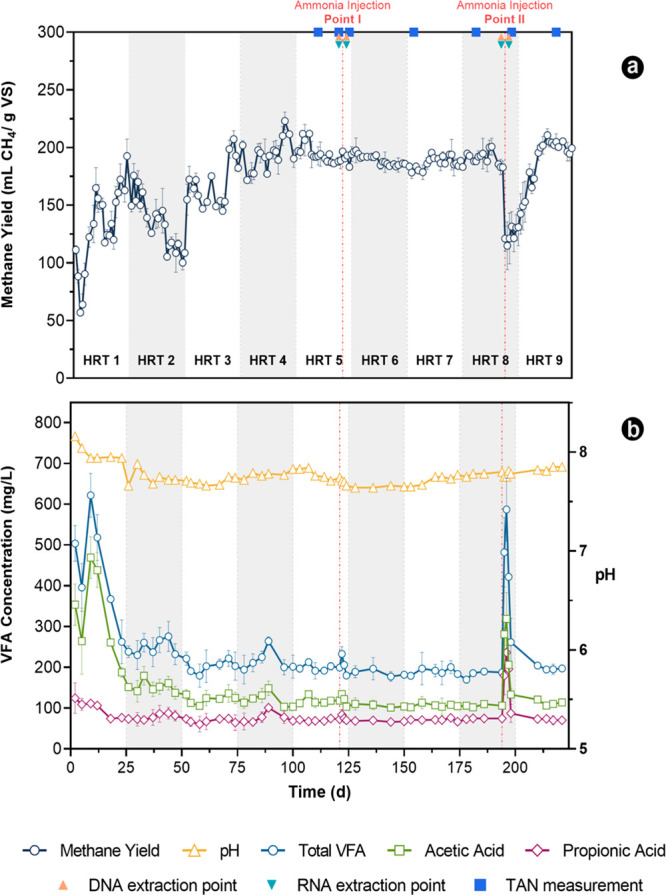
Reactor performance with respect to (a) methane yield and (b) VFA concentration and pH. Each point is the mean value of the three bioreactors (therefore, the standard deviation is provided). The HRT of each cycle was 25 days. The red vertical dashed lines indicate the time of the ammonia injection. DNA extraction points are denoted by orange triangles, RNA extraction points by green inverted triangles, and TAN measurements by blue squares.

The reactors were in operation for 224 days, during which the essential biochemical parameters methane yield, pH levels, and VFA concentration were continuously monitored (Figure 1a, b). Among the VFA, acetate and propionate were the major intermediates detected and, for this reason, were the only ones, along with the total VFA (TVFA), considered for discussion (Figure 1b). The performance of the reactors in terms of the methane yield is depicted in Figure 1a. The system needed approximately four HRTs (106 days) to stabilize its methane production. Subsequently, the daily fluctuations in reactor performance remained consistently below 10%, aligning with the conventional practice used to identify a state of steady-state operation in anaerobic digesters.^37^ Once steady-state operation was established, no significant variations were observed among the three reactors, indicating that they performed comparably, producing an average of 190 ± 10 mL CH_4_/gVS. On day 122 of operation, a single pulse disturbance was introduced into the reactors by injecting 1.5 g NH_4_^+^/L_R_, resulting in an average FAN concentration of 86 ± 12 mg NH_3_–N/L_R_. On initial observation, the injection did not appear to cause process imbalance, as evidenced by the constant methane yield (Figure 1a). However, from a biochemical point of view, the system did not remain unperturbed, and a modest increase was detected in the concentration of VFA, i.e. 233 ± 18 mg of TVFA compared to the average value of 188 ± 8 mg of TVFA under the steady-state period (Figure 1b). This result suggests that ammonia influenced the microbiota and is supported by the transcriptional activity of the methanogens, as will be discussed below. Despite this mild impact, the biogas production was not affected, indicating that the microbes were resilient enough to quickly adapt and recover.

Although the initial shock load was of low intensity, it was expected to influence the dynamics of the microbial populations. For this reason, a period of approximately three HRTs (days 123–193) was implemented to allow for the community’s equilibration prior to the second ammonia injection. On day 194, a single pulse of 5 g of NH_4_^+^/L_R_ was imposed into the reactors, causing an immediate rise in FAN levels to 200 ± 16 mg of NH_3_–N/L_R_. This addition noticeably affected the operation, as demonstrated by the 41% reduction in the methane yield on day 196 (Figure 1a), considering the steady-state average value of 190 mL of CH_4_/gVS as a baseline. Thereupon, despite the strong perturbation, the performance of the reactors returned to prior to the shock stable operation after a “lag period” of 14 days, as evidenced by the stabilization of the methane yield (Figure 1a). The process disturbance corresponded with the accumulation of VFA (Figure 1b), exhibiting a sharp rise in the concentration following the shock. The highest concentration of the average TVFA was observed on day 196, reaching 587 ± 137 mg/L. Acetate was the most dominant VFA having a value equal to 318 ± 65 mg/L, followed by propionate at 236 ± 58 mg/L. Similar outcomes were also reported in a previous study, showing that acetate is the primary type of VFA that accumulates during ammonia inhibition rather than propionate.^38^ The observed value did not exceed the critical threshold for reactor operation, as defined by Angelidaki et al. (2005), who determined 1.5 g/L TVFA as a benchmark for a well-functioning AD process.^39^ Furthermore, the observed increase in VFA concentration was temporary, as all reactors on day 210 returned to the status of the preshock phase (i.e., the same concentration levels recorded between days 124 and 194). The pH values of all reactors remained relatively stable throughout the entire experimental period, ranging from 7.63 to 8.16 (Figure 1b), within the optimal for AD. Considering that ammonia can cause pH fluctuations, particularly by increasing the pH due to its ionization,^40^ cattle manure appeared effective in preventing this change that could have negatively impacted the process. This efficacy is attributed to the high buffer capacity of cattle manure, which protects the process from disturbances caused by pH fluctuations.^41^

### 3.2 Metagenomic Profiling of the Community

**Figure 2:**
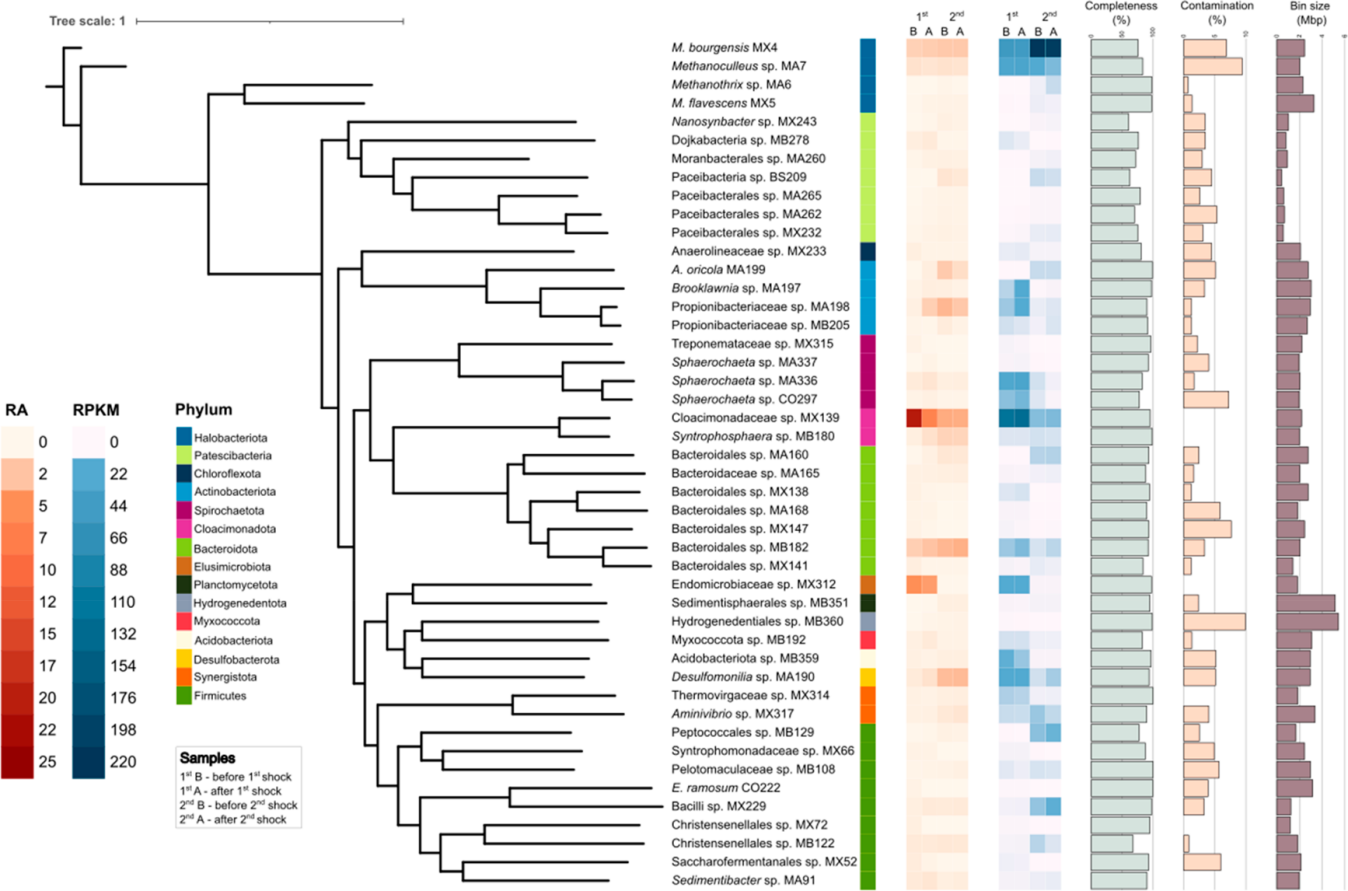
Overview of the identified microbial taxa. Completeness, contamination, and bin size of the corresponding MAGs, and average RA and RPKM across the samples. Here are reported only the taxa with a RA of 0.5 or higher in at least one sample. (“B” stands for before shock, while “A” stands for after shock).

The assembly and binning resulted in 364 MAGs, of which 163 were of high quality. Given the high complexity of the community, only the 46 MAGs with an RA of 0.5% or higher in at least one sample will be considered for detailed inspection (Figure 2). Within this group, four archaea are worth noticing: *Methanoculleus bourgensis* MX4, *Methanoculleus* sp. MA7, *Methanothrix* sp. MA6, and *Methanosarcina flavescens* MX5. The persistent dominance of the two *Methanoculleus* spp. confirms the preference for the hydrogenotrophic pathway in high ammonia concentrations, aligning with the typically greater ammonia tolerance of hydrogenotrophic methanogens.^6^ Nonetheless, *M. flavescens* MX5, a known versatile methanogen,^42^ and *Methanothrix* sp. MA6, suggested to produce CH_4_ through CO_2_ reduction with direct interspecies electron transfer (DIET) in addition to the acetoclastic pathway,^43^ demonstrated a rise in RA after the first shock event (Table S3). As will be subsequently discussed, this increase implies that these species potentially altered their metabolic pathways to ensure survival. Conversely, it cannot be disputed that the metabolism shift could be further reinforced by the competitive assimilation of acetate. The preference for the hydrogenotrophic pathway could be enhanced by the presence of syntrophic acetate-oxidizing bacteria (SAOB) belonging to the family Syntrophomonadaceae,^44^ such as Syntrophomonadaceae sp. MX66. These bacteria can potentially release H_2_ and CO_2_ from acetate via the reductive acetyl-coA pathway, also known as the Wood-Ljungdahl pathway. While responsible for synthesis of acetyl-coA from CO_2_ in homoacetogenic bacteria, it is believed to act in the opposite (oxidative) direction in SAOB, therefore providing substrates for hydrogenotrophic methanogens, possibly in combination with the Glycine Cleavage System.^45^

Starting from the initial complex microbial community, the results of RA and RPKM (Figure 2) showed how several species were led to extinction after the first shock, probably due to their inability to tolerate ammonia (e.g., Endomicrobiaceae sp. MX312). These MAGs were probably replaced in their metabolic roles by other microbes that proliferate, such as *Desulfomonilia* sp. MA190, *Syntrophosphaera* sp. MB18, and *Aminivibrio* sp. MX317, all increasing substantially (avg log_2_FC 0.51) but not significantly (*p*-value > 0.05) in their RA (Figure 2, Supplementary table S3). On the other hand, microbes that survived the first shock generally remained stable, such as the two *Methanoculleus* spp., while others experienced a severe drop as *Cloacimonadaceae* sp. MX139, *Sphaerochaeta* sp. MA336, and *Sedimentibacter* sp. MA91 (Figure 2). The impact of the second shock was much stronger, and some species (e.g., Endomicrobiaceae MA312) were not capable of withstanding the new conditions. However, several bacteria which resisted the first shock managed to either increase in abundance (e.g., *Desulfomonilia* sp. MA190, *Aminivibrio* sp. MX317, and Bacteroidales sp. MB182) or to remain almost stable (e.g., Christensenellales sp. MB122). Notably, none of them is known for their ability to process N-rich compounds according to the literature; however, their survival must also be associated with some metabolic resistance ability, including synthesis of osmoprotectant and H^+^ replenishment.^46^ Among the archaeal species, only *M. bourgensis* MX4 and *Methanoculleus* sp. MA7 appeared to tolerate the higher ammonia concentrations, while a tendency of decreased RA was observed for both *Methanothrix* sp. MA6 and *M. flavescens* MX5 (Figure 2, Table S3). Furthermore, at higher ammonia concentrations an increased presence of syntrophic propionate-oxidizing bacteria (SPOB) from the genus *Syntrophosphaera*(47) and the family Pelotomaculaceae^48^ was registered. Notably, both *Syntrophosphaera* sp. MB180 and Pelotomaculaceae sp. MB108 exhibited an increase in their RA, although it was not significant (Supplementary table S3).

Overall, the exposure to the first shock (i.e., 1.5 g of NH_4_–N/L and FAN 86 ± 12 mg of NH_3_–N/L_R_) potentially initiated a selective process within the microbial community. This could help the development of a more resilient community capable of tolerating elevated ammonia levels. To support this hypothesis, the impact of two ammonia shocks was also investigated at the transcriptional level. This aimed to delve deeper into the transcriptional activity and to link these observations to the metagenomic analysis findings. In particular, the impact on the methanogenesis of the dominant archaea and the regulation of cellular homeostasis in response to high concentrations of ammonia was evaluated.

### 3.3 Effect of Ammonia on the Methanogenic Microcosm

**Figure 3:**
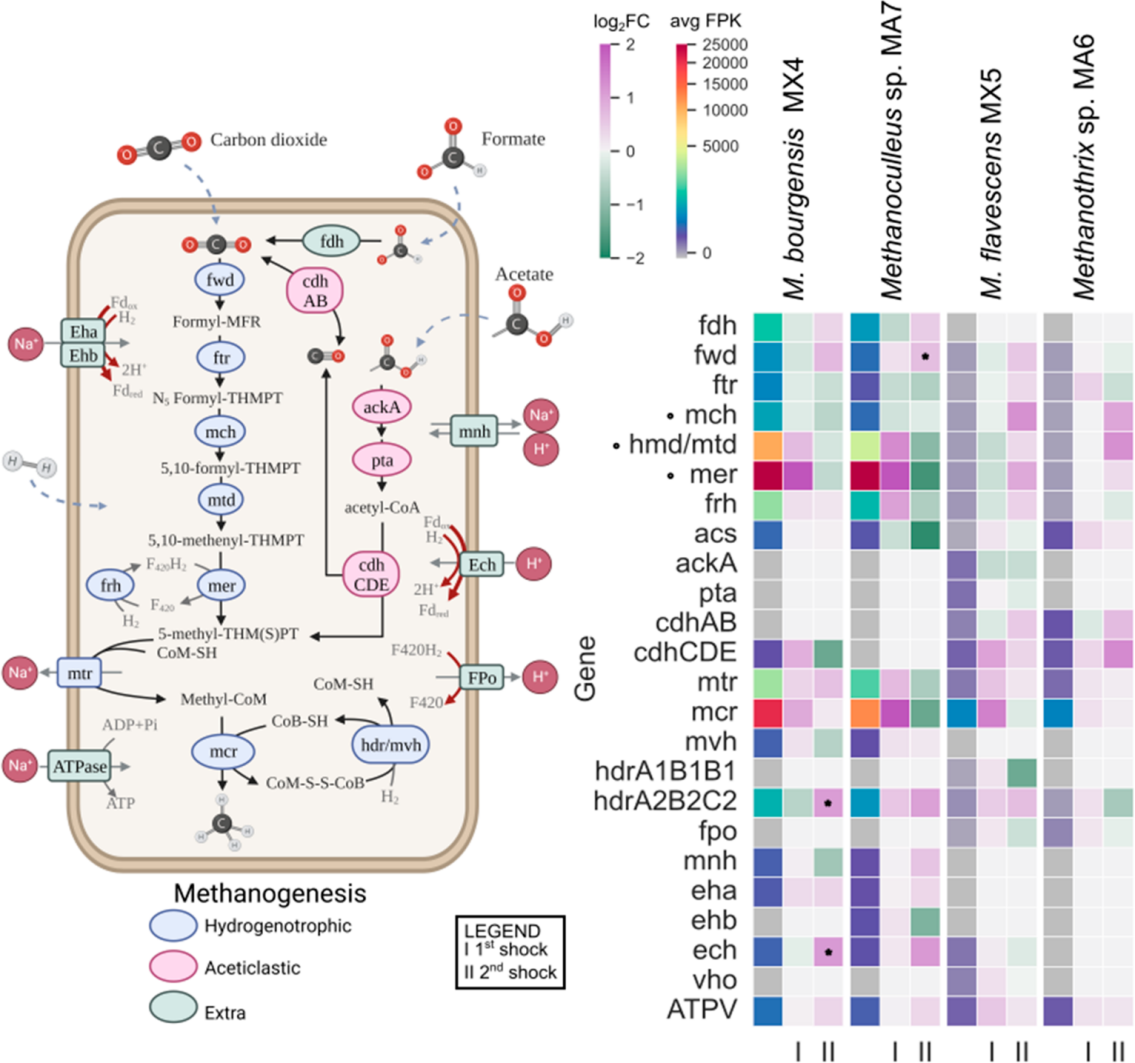
Methanogenic activity of archaeal MAGs and differential expression under the two shocks. (A) Metabolic reconstruction of methanogenesis and carbon assimilation. (B) Expression of genes for methanogenesis, carbon assimilation, and energy conservation, with the outcome of differential expression analysis: average FPK and log_2_FC by enzymes are reported as described in Materials and Methods. The symbol “I” denotes the log_2_FC before and after the initial shock, whereas “II” is used to signify the log_2_FC before and after the subsequent shock. Genes highlighted with “°” were reconstructed for *M. bourgensis* MX4 using the reference genome of the isolate BA1 (RefSeq, GCF_900036045.1) deposited at the NCBI. Genes in the average FPK heatmap not present or reconstructed in the analyzed MAGs were represented with the color gray. Enzymes with at least one differentially expressed gene with *p*-value <0.05 are marked with *.

The correlation analysis between the biochemical parameters and the microbial activity revealed that CH_4_ was the factor explaining most of the microbial abundance variability (Table S4), with a high Spearman correlation (≥0.88) (Table S5). Hence, a closer examination of individual archaeal MAGs and of the pathways associated with methanogenesis was conducted to gain deeper insight into the adaptation and the contribution of each shock (Figure 3). The changes in the expression levels of the genes during both shock loads are represented as log_2_FC. MAGs assigned to the genus *Methanoculleus* (MX4 and MA7) exhibited comparable gene expression responses, with notable distinctions in *mtr* and *mcr* genes. Likewise, *M. flavescens* MX5 and *Methanothrix* sp. MA6 demonstrated a similar pattern in the regulation of genes in response to the shocks. When subjected to increased NH_4_^+^ concentrations during the second pulse (5 g NH_4_^+^/L_R_), an apparent shift in the transcriptional response of genes associated with the acetoclastic pathway was observed. Specifically, genes encoding subunits *cdhCDE* of the carbon monoxide dehydrogenase/acetyl-CoA synthase (CODH/ACS) complex in *M. flavescens* MX5 displayed a weaker upregulation following shock II as compared to shock I, with log_2_FC values of 0.39 and 1.03, respectively (Figure 3), although neither shock led to statistically significant changes in expression of these genes. This complex has a central role in the decomposition of Acetyl-CoA, oxidizing the carbonyl group to CO_2_ and transferring the methyl group to tetrahydrosarcinapterin (THSPT).^49,50^*M. flavescens* MX5 also downregulated the genes involved in the *ackA*-*pta* pathway after the second shock, employed by members of the *Methanosarcina* genus to interconvert acetate to Acetyl-CoA.^51^ The reduction of acetate in *Methanothrix* spp. is catalyzed by the Acetyl-CoA synthetase (*acs*),^52^ whose encoding gene showed no change in expression level after the two shocks in *Methanothrix* sp. MA6. On the contrary, data suggested that the two acetoclastic archaea might upregulate genes related to the CO_2_ reduction pathway following shock II (Figure 3), despite not achieving statistical significance. These included genes encoding enzymes such as tetrahydromethanopterin S-methyltransferase (*mer*), methylenetetrahydromethanopterin dehydrogenase (*mtd*), and methenyltetrahydromethanopterin cyclohydrolase (*mch*). Based on the previous observations, it is reasonable to assume that the ammonia loading significantly influenced the methanogenic activity of these two MAGs, inducing a change from the acetoclastic to the CO_2_ reduction route. The immediate shift, observed within 14 h after the addition of NH_4_^+^, can be linked to thermodynamic reasons since the transition from the acetoclastic to the CO_2_ reduction pathway can result in increased energy output. It is known that when NH_3_ enters the cell, it competes with endoenzymes for protons that are required to produce reducing power.^53^ As the energy reservoir of the cell constantly decreases, the cell switches to the hydrogenotrophic pathway, which provides more energy than the acetoclastic one as a survival mechanism.^54^ However, this shift needs to be intended as a sole mechanism of survival employed by both *M. flavescens* MX5 and *Methanothrix* sp. MA6 with the purpose of reducing the energy demand of their anabolic metabolism, particularly in the context of methanogenesis. Both the RA and the expression levels of all genes, including both hydrogenotrophic and non-hydrogenotrophic ones, in the two acetoclastic archaea are significantly lower compared to those of the two *Methanoculleus* species. As a result, both *M. flavescens* MX5 and *Methanothrix* sp. MA6 is likely to make a lesser contribution to methane production within the microbial community under investigation.

Concerning the two *Methanoculleus* spp. MAGs, both appeared to downregulate the hydrogenotrophic pathway in response to shock II, although without statistical significance (Figure 3). In *Methanoculleus* sp. MA7, calculated log_2_FC for genes *ftr*, *mcr*, *mer*, *mtd*, and *mtr* ranged between −0.65 and −1.70 (Table S8). In particular, *mcr* genes demonstrated downregulation after shock II, according to the associated log_2_FC value of −1.37. The expression of *mcr* has been linked to either methane production rate or methane yield and has been suggested as a reliable biomarker for ammonia inhibition due to its high consistency.^55^ Subsequently, the decrease in *mcr* expression would explain the considerable reduction in methane detected during shock II. Notably, this effect appeared to differ from shock I, as many backbone genes of the pathway displayed relatively large positive log_2_FC after shock I but a negative log_2_FC after shock II. *M. bourgensis* MX4 displayed trends similar to those of *Methanoculleus* sp. MA7, although less pronounced (Table S8). In particular, the log_2_FC of *mcr* in this MAG in shock II was still positive, although lower than that in shock I. Overall, the higher ammonium load in shock II appeared to induce a reduced expression of the hydrogenotrophic pathway in the two dominant hydrogenotrophic methanogens. These observations were based on the calculated log_2_FC values but did not exhibit statistical significance.

Of particular interest is the transcriptional activity of the MAGs encoding energy conservation complexes since these genes have been proposed to contribute to the survival of the methanogens in environments with high ammonia levels.^56^ Metagenomics revealed the presence of enzymes linked to the Eha complex, whose genes (*ehaA-T*) were upregulated in both *M. bourgensis* MX4 and *Methanoculleus* sp. MA7 following the second shock (Figure 3). This membrane-bound energy-converting hydrogenase is considered essential for its anaplerotic role,^57^ coupling its action with the Fwd, to reduce a ferredoxin and translocate Na^+^. However, despite replenishing the intermediates lost to leaky electron bifurcation or biosynthesis and anabolism, the electron transfer mediated by Eha does not result in a net energy gain through methanogenesis.^57^ To separate the electron pool utilized for anabolism from that used to replenish methanogenesis intermediates, an alternative mechanism has recently been proposed.^58,59^ In both *Methanoculleus* spp. an upregulation of genes encoding for Hdr, Fdh and Fwd was detected as statistically significant (*p* < 0.05) (Figure 3). These three enzymes are known to contribute to the formation of a bifurcating multienzyme complex, enabling direct electron flow from formate to the heterodisulfide bond,^58^ and thus, their coordinated regulation is expected. More specifically, the Hdr/Fwd cluster mediated electron transfer through electron bifurcation, resulting in a net energy gain from methanogenesis. Frh is proposed to replace the function of Mvh, producing the necessary F420H_2_ and thus reducing the energy cost of the methanogenesis process, as observed in previous studies on *Methanocorpusculum parvum*(58) and *Methanococcus maripaludis*.^59^ Furthermore, a second energy-converting hydrogenase complex was found to be upregulated after the second shock. Specifically, the genes encoding the ferredoxin-dependent Ech hydrogenase (*echA-F*) exhibited increased expression (*p* < 0.05) in both *M. bourgensis* MX4 and *Methanoculleus* sp. MA7, with log_2_FC values of 1.13 (II) and 1.15 (II), respectively (Figure 3). The Ech hydrogenase has been previously characterized in several methanogenic archaea under ammonia stress, i.e., *echC* in *Methanosarcina barkeri*,^60^ and is associated with the oxidation of reduced ferredoxin and the release of H_2_.^61^ Rather than a direct effect, the restored proton motive force resulting from Ech activity embodies an indirect energy conservation strategy that plays a key role in the early stages following an osmotic shock. Its activity is coupled with proton translocation, aiding in the re-establishment of the basal proton gradient required by the ATPase for ATP synthesis.^62^ Hence, this overall upregulation of energy-converting enzymes could also be hypothesized as a survival mechanism for archaea belonging to the *Methanoculleus* genus.^63^

### 3.4 Effect of Ammonia on the Cellular Osmotic Balance

**Figure 4:**
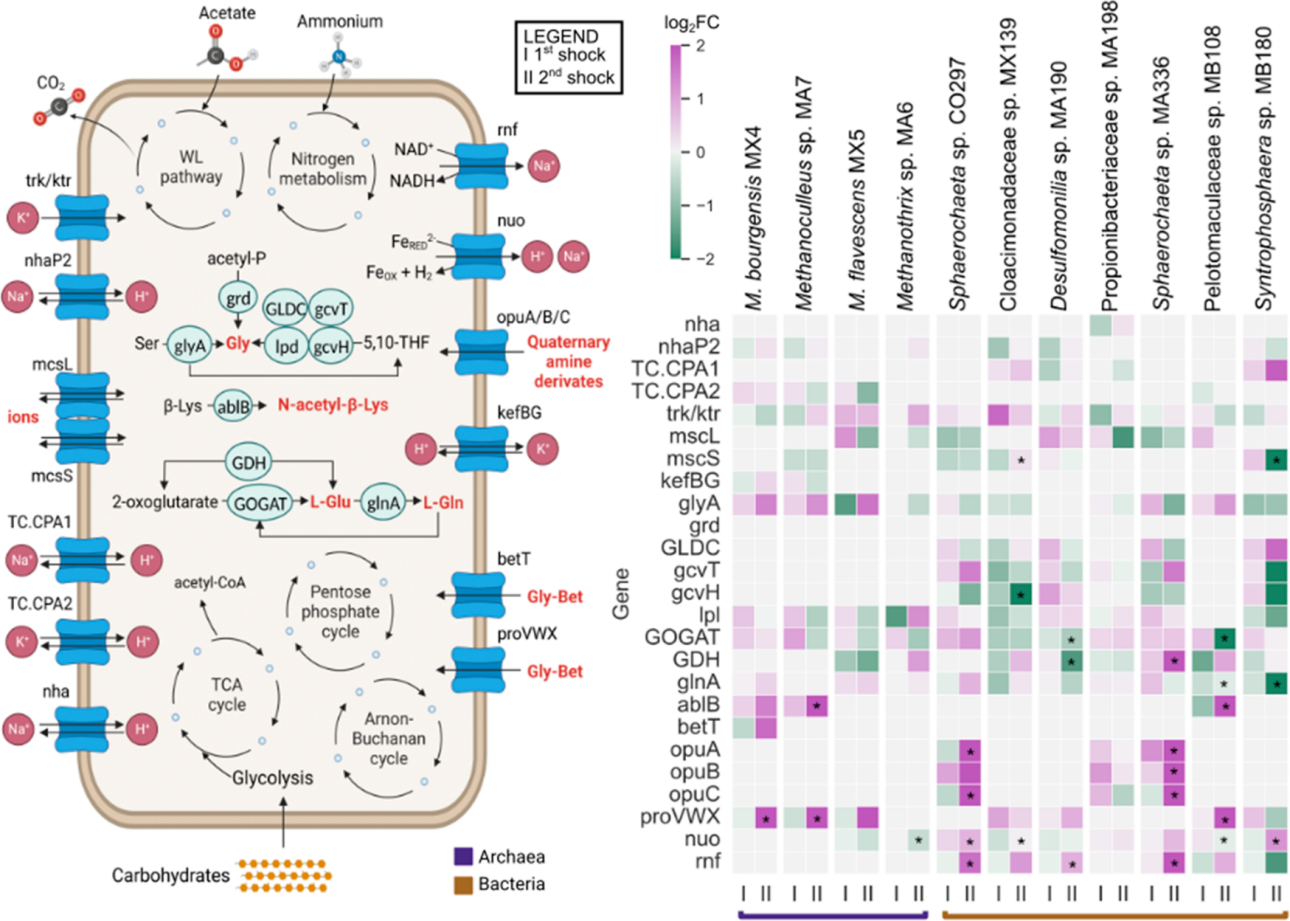
Homeostatic regulation mediated by osmoprotectant ion/solute synthesis and activation of cation balance mechanisms. (A) Metabolic reconstruction of the microbial metabolism in response to ammonia. (B) Expression of genes involved in ion transport and in the osmoprotectants biosynthesis. The outcome of differential expression analysis (log_2_FC) is reported in color and analysis was performed as described in the Materials and Methods. The symbol “I” denotes the log_2_FC before and after the initial shock, whereas “II” is used to signify the log_2_FC before and after the subsequent shock. Enzymes with at least one differentially expressed gene with *p*-value <0.05 are marked with *.

While previous studies have examined the adaptation of the microbiome to increasing levels of ammonia, the mRNA-level response, particularly regarding mechanisms involved in the recovery from ammonia-induced stress, remains poorly understood. More into detail, integrated metagenomics and metaproteomics focused only on elucidating the impact of ammonia on methanogens and syntrophs, as well as activity of key enzymes during AD.^64,65^ However, upregulation of enzymes under ammonia-stressed conditions, potentially leading to a mechanism for controlling ammonia toxicity and stabilizing the AD process, is still largely unexplored. Therefore, it is important to use genome-centric metatranscriptomics to map the early response of the microbiota, which can aid in understanding and managing the turn-on and turn-off steps of the AD process. These efforts would facilitate an understanding of strategies to augment the resilience of active microbes and enzymes against ammonia. Through this achievement, recovery and even promotion of the reaction rate of the key steps being regulated by the ammonia-inhibiting enzymes becomes possible. According to existing literature, FAN entering the cell is converted to ammonium through protonation,^66^ which can lead to temporary proton imbalance, potassium deficiency, and strong osmotic stress.^67^ More specifically, as soon as the NH_4_^+^ concentration rises, the cells respond with K^+^ efflux;^68,69^ therefore, the expression of genes involved in the K^+^ transport systems was further investigated. The Trk/Ktr potassium uptake (*trkA*, *trkH*) and the glutathione-regulated potassium-efflux systems (*kefB*, *kefG*) were both upregulated after the second shocks in several MAGs (Figure 4). It is worth noting that the two *Methanoculleus* spp. exhibited distinct behaviors and yet managed to survive the second shock. In *M. bourgensis* MX4 there was an increased expression of the trk/ktr system, whereas *Methanoculleus sp. MA7* upregulated the KefB efflux system (Table S9). Conversely, the expression of mechanosensitive channels (*mscL*, *mscS*) varied depending on the microbes. These channels are generally activated when the cell experiences increased turgor pressure,^70^ so we can expect that MAGs upregulating them were experiencing an osmotic downshock, like in Cloacimonadaceae sp. MX139 (*p* < 0.05) and *Desulfomonilia* sp. MA190 (Figure 4).

The effects of the decrease in intracellular protons due to the high NH_4_^+^ level were also evaluated. Cells can replenish the depleted protons through the activity of various antiporters, including Na^+^:H^+^ antiporter (*nha, nhaP2*)^71^ and cation:H^+^ antiporter such as those belonging to the CPA1 and CPA2 families (e.g., *TC.CPA1*, *TC.KEF*).^72^ The CPA1 antiporter was exclusively upregulated in *M. bourgensis* MX4, while the CPA2 family was upregulated in both *Methanoculleus* sp. MA7 and *Syntrophosphaera* sp. MB180. This enhanced activity likely played a crucial role in restoring the H^+^ gradient required for ATPase activity, thereby favoring the survival of these two hydrogenotrophic archaea in comparison to *M. flavescens* MX5 and *Methanothrix* sp. MA6. The proton flow is also supported by energy conservation mechanisms in the microbiome, such as electron-bifurcating flavoprotein complexes (Rnf, Ech)^73^ and membrane-bound NADH-ubiquinone oxidoreductase (Nuo).^74^ After the second shock, an upregulation of the genes encoding these complexes (*rnf*, *nuo)* was detected as statistically significant (*p* < 0.05) in different microbes, such as Pelotomaculaceae sp. MB108, *Desulfomonilia* sp. MA190, *Syntrophosphaera* sp. MB180, and the two *Sphaerochaeta* species (Figure 4). Furthermore, the enhanced expression of Ech and Eha hydrogenases in both *Methanoculleus* spp. after shock II might have contributed to the restoration of their basal proton force through H_2_-dependent reduction of ferredoxin coupled with reverse electron transport, resulting in increased energy efficiency.^57,75^ The restored proton force coupled with the higher expression of the *mnh* operon by *Methanoculleus* sp. MA6 (Figure 3) results in the translation of membrane transport proteins that catalyze the efflux of cytoplasmic sodium, potassium, or lithium ions in exchange for external hydrogen ions.^76^

The last mechanism that microorganisms implement to achieve osmotic balance and counteract ammonia inhibition is the synthesis and transport of osmoprotectants (glutamine, glycine, glycine/betaine, glutamate and N^ε^-acetyl-l-lysine) and small organic molecules that act as osmolytes.^77^ The genes encoding enzymes involved in the biosynthesis of glycine (*glyA*)^78^ and glutamine (*glnA*)^79^ were upregulated in several MAGs, including *M. bourgensis* MX4, Pelotomaculaceae sp. MB108, *Sphaerochaeta* sp. CO297 and Cloacimonadaceae sp. MX139 (Figure 4). Glutamate is another known compatible solute, whose synthesis can be catalyzed by either the glutamate synthase (*GOGAT*)^80^ or the glutamate dehydrogenase (*GDH*) in the reverse direction,^81^ both upregulated in the previously mentioned MAGs (Figure 4). Moreover, glutamate synthesis was also upregulated in *Methanoculleus* sp. MA7 and *Sphaerochaeta* sp. MA336 (*p* < 0.05), further highlighting the key role of this compound. Another gene displaying a key role, especially in the two *Methanoculleus* spp., is *ablB*,^82^ encoding the beta-lysine N6-acetyltransferase responsible for the synthesis of N^ε^-acetyl-l-lysine under salt stress conditions. The *ablB* gene was absent in the acetoclastic archaea but showed upregulation in both *M. bourgensis* MX4 and *Methanoculleus* sp. MA6 (*p* < 0.05), as well as in the putative syntrophic Pelotomaculaceae sp. MB108 (*p* < 0.05). The activity of *ablB* was only documented in *Methanothrix* species until now,^52^ thus this is the first time that synthesis of Nε-acetyl-l-lysine was demonstrated also in hydrogenotrophic methanogens.

The transport of compatible solutes was also significantly upregulated in several MAGs, with a notable preference for the import of glycine betaine from the environment through three distinct systems. The expression of the ABC transporter for glycine betaine ProVWX^83^ was enhanced and statistically significant (*p* < 0.05) in the two *Methanoculleus* spp. and in the putative syntrophic Pelotomaculaceae sp. MB108 (Figure 4), highlighting the importance of this osmoprotectant in the rapid response to unbalanced osmotic conditions. While the *proVWX* genes were widespread among the microbial community under investigation, *M. bourgensis* MX4 was the only MAG upregulating a second transporter for glycine betaine (BetT). This transporter, belonging to the BCCT family, is responsible for the exogenous glycine betaine import from the environment^84^ and is notably abundant in stressful ecosystems like Antarctica.^85^ Lastly, in the two *Sphaerochaeta* spp. the osmostress-protective effect was achieved through the expression of the opu operons (*opuA*, *opuB*, *opuC*)^86^ after the second shock (*p* < 0.05). The OpuA, OpuB, and OpuC transporters are members of the binding-protein-dependent ABC superfamily that use the hydrolysis of ATP to energetically fuel the overall transport process, especially in stressful conditions (e.g., increase/decrease in osmolarity) as previously reported for *Bacillus subtilis*.^86^ Altogether, these results confirm and highlight the key role of compatible solutes in allowing microbial survival at a high osmolarity. The level of gene expression associated with such compatible solutes is increased in the microbiome right after ammonia exposure. Specifically, the MAGs upregulating these genes remained more stable in RA both within and between the shocks, since they were able to react to the stress and cope with the new cellular conditions.

Methanogens that possess and upregulate multiple energy-converting hydrogenases (Ech, Eha, Ehb), such as the two *Methanoculleus* species, may be more energy-efficient and able to thrive better than those without these complexes.^46^ Furthermore, the upregulation of genes involved in the synthesis and transport of osmoprotectants, which are specific to hydrogenotrophic archaea (*nhaP2*, *ablB*, and *betT*), can explain their higher tolerance to elevated ammonia concentrations compared to acetoclastic species (Figure 4). Transcriptomic results indicate that the ammonia shock had a greater effect on gene expression in shock II. This outcome supports the expectation that the microbiome would be more influenced during this phase due to the higher ammonia concentration that was induced inside the reactors.

In brief, this study aimed to clarify the microbial activity using a comprehensive approach combining biochemical and genomic data. Although shock “I” did not visibly affect methane production, monitoring VFA concentrations proved to be a reliable indicator of even minor system disturbances. This highlights the value of monitoring VFA concentrations in practical applications as an early warning signal of process disruptions, offering a more actionable metric than merely observing biogas production or composition. Regarding the methanogenic population, of particular interest is the indication that the versatile methanogens *Methanothrix* sp. MA6 and *M. flavescens* MX5 might enact a metabolic switch from the acetoclastic pathway during the second shock, possibly choosing a thermodynamically more favorable route as a survival mechanism. Although stimulating, these findings will require further exploration and confirmation. Furthermore, the transcriptomic analysis revealed the upregulation of numerous genes associated with osmotic balance, which act as a survival mechanism for the microbial cells. These mechanisms included the activation of mechanosensitive channels (MscL, MscS), indicating increased turgor pressure within the cells and potassium uptake through Tkr/Ktr system. Additionally, the activation of antiporters facilitated the replenishment of protons, while the synthesis and transport of osmoprotectants (such as glycine/betaine and Nε-acetyl-l-lysine) and other compatible solutes played a crucial role in the cell’s homeostasis. Overall, the outcomes of the present study highlight that the profound understanding of the microbial community’s architecture and activity gives valuable information and can assist in the design of practical operational strategies or the potential development of diagnostic tools for full-scale biogas plants. For instance, insights from the microbial dynamics illuminate the importance of tailoring feedstock compositions based on microbial tolerance in certain stressors, adjusting process parameters such as pH and temperature in relation to ammonia concentration, harnessing the potential of diagnostic biomarkers (evidenced by *mcr* expression associated with ammonia inhibition), and establishing informed protocols for digester recovery in the aftermath of disturbances caused by increased ammonia levels.

## Supporting Information Available

Information about the binning process, general insights into microbiota activity, and additional figures (S1–S3) (PDF)
Additional tables (S1–S9) containing information on biochemical characteristics, total and free ammonia levels, statistical information, and additional information about the metagenomic and metatranscriptomic analyses (XLSX)
