# Supplementary material for: Decoding Microbial Responses to Ammonia Shock Loads in Biogas Reactors through Metagenomics and Metatranscriptomics
Source: Environ Sci Technol. 2023 Dec 19;58(1):591–602. doi: 10.1021/acs.est.3c07840 (PMC10785759; doi:10.1021/acs.est.3c07840)
Supplement: Supplementary file 1 — es3c07840_si_001.pdf [file es3c07840_si_001.pdf]

# Decoding microbial responses to ammonia shock loads in biogas reactors through metagenomics and metatranscriptomics

Maria Gaspari[a,b+], Gabriele Ghiotto[c+], Victor Borin Centurion[c], Thomas Kotsopoulos[b], Davide Santinello[c], Stefano Campanaro[c*], Laura Treu[c§], Panagiotis G. Kougias[a*§].

[a]Soil and Water Resources Institute, Hellenic Agricultural Organisation Dimitra, Thermi, Thessaloniki 57001, Greece

[b]Department of Hydraulics, Soil Science and Agricultural Engineering, School of Agriculture, Aristotle University of Thessaloniki, Thessaloniki 54124, Greece

[c]Department of Biology, University of Padova, Padova 35121, Italy

[+]These authors contributed equally to the work; [§] equal contribution

*Corresponding authors: stefano.campanaro@unipd.it and p.kougias@swri.gr

**SUMMARY OF CONTENTS**

Number of Pages: 6

Number of Figues: 3

**TABLE OF CONTENTS**

Section S1: Binning information . . . . . . . . . . . . . . . . . . . . . . . . . . . . . . . . . . . . . . . . . S2

Section S2: General insight into microbiota activity . . . . . . . . . . . . . . . . . . . . . . . . . S2

References . . . . . . . . . . . . . . . . . . . . . . . . . . . . . . . . . . . . . . . . . . . . . . . . . . . . . S3

Supplementary Figure S1: Module completeness . . . . . . . . . . . . . . . . . . . . . . . . . . . . S4

Supplementary Figure S2. Metabolic activity of the most abundant MAGs . . . . . . . . S5

Supplementary Figure S3. Physicochemical vs metagenomics and metatranscriptomics genome abundance data . . . . . . . . . . . . . . . . . . . . . . . . . . . . . . . . . . . . . . . . . . . . . S6

## Section S1: Binning information

The average percentage of reads alignment on the assembly was 92.04%, meaning that the microbial community was largely represented, with 61.78% of sequences aligned to the medium-high quality MAGs (from 53.46% to 67.84%, depending on the sample). This indicates that results showed a reliable representation of the system and depicted most of the microbial species. Regarding the taxonomic analysis, the microbial community was composed of 17 Archaea (4.67%) and 347 Bacteria (95.33%), and the most represented phyla were Firmicutes (39.01%), Bacteroidota (12.64%) and Patescibacteria (7.14%).

## Section S2: General insight into microbiota activity

Community-level pathway analysis was performed to examine the metatranscriptomic changes that occurred because of the two $NH_4^+$ shocks. The analysis involved calculating the average FPK of genes within each KEGG module of interest. The changes in expression levels of the genes and modules involved in energy and carbohydrate-related metabolic pathways were examined. The findings revealed that the methanogenesis pathway (M00357, M00567) was the metabolic route with the highest expression level, especially for the two *Methanoculleus* species (**Supplementary Figure S2**).

As expected, the overall efficiency of the AD process was affected by ammonia addition, as already reported in the literature[1]. The impact of high ammonia concentrations is supported by the PCA analysis **(Supplementary Figure S3)**, which reports a significant association (p-value 0.04) between methane concentration and the microbial activity of the first shock while showing an inverse correlation to the second shock **(Supplementary Table S4)**. Notably, several pathways associated with carbon metabolism, including Glycolysis/Gluconeogenesis and Propanoate metabolism (M00013, M00741), displayed an overall low expression level (below 400 FPK), suggesting that acetogenic archaea and acetate-utilizing microbes were also negatively impacted. It is well-documented that methanogens predominantly depend on gluconeogenesis for the synthesis of carbohydrates, such as pentoses and hexoses[2] and that that certain microbes that utilize acetate adopt the Embden-Meyerhof-Parnas [EMP] pathway in an anabolic direction, specifically for gluconeogenesis[3]. Propionate-degrading

species members of the Cloacimonadaceae[4] family, such as Cloacimonadaceae sp. MX139, showed low activity (below 300 FPK) of crucial functional modules, including M00374, M00375 and M00376 (**Supplementary Figure S2**), which may contribute to the observed propionate accumulation following the two shocks (**Figure 1b**). The fluctuations in acetate concentrations recorded after the two ammonia shocks could also be determined by the elevated expression level of the Wood-Ljungdahl pathway (M00377), which is associated with the conversion of acetate to $CO_2$. The putative acetate oxidation activity of Pelotomaculaceae sp. MB108 and Bacteroidales sp. MB182, increasing after both shocks in the marker gene *fhs* ($log_2FC$ of average FPK of 0.71 and 0.92 respectively), can result in the generation of $H_2$ and $CO_2$ needed by the methanogenic archaea to perform hydrogenotrophic methanogenesis and recover from the inhibited state. Lastly, ATP-synthesis related modules had low expression profiles, for example changing from 233 to 127 FPK after the second shock in *Sphaerochaeta* sp. MA336, confirming how energy production is negatively affected by the ammonia concentration within the cell. Notably, the expression levels of both V-type ATPase (M00159) and NADH:quinone oxidoreductase (M00144) modules were higher in the microbes that exhibited a high degree of transcriptional activity, such as the four methanogenic archaea, and the propionate-degrading Cloacimonadaceae sp. MX139 (**Supplementary Figure S2**).

Overall, this initial investigation was focused on the KEGG modules and provided a general indication of the metabolic traits present in the individual microbes. However, by scrutinizing the data at a higher level of detail, we can move beyond the rough expression analysis and gain a more nuanced and specific understanding of the gene expression changes triggered by the ammonia shock.

**References**

(1) Kougias, P. G.; Angelidaki, I. Biogas and Its Opportunities—A Review. *Front. Environ. Sci. Eng.* **2018**, *12* (3), 14. https://doi.org/10.1007/s11783-018-1037-8.

(2) Bräsen, C.; Esser, D.; Rauch, B.; Siebers, B. Carbohydrate Metabolism in Archaea: Current Insights into Unusual Enzymes and Pathways and Their Regulation. *Microbiol. Mol. Biol. Rev. MMBR* **2014**, *78* (1), 89–175. https://doi.org/10.1128/MMBR.00041-13.

(3) Frank, J. A.; Arntzen, M. Ø.; Sun, L.; Hagen, L. H.; McHardy, A. C.; Horn, S. J.; Eijsink, V. G. H.; Schnürer, A.; Pope, P. B. Novel Syntrophic Populations Dominate an Ammonia-Tolerant Methanogenic Microbiome. *mSystems* **2016**, *1* (5), 10.1128/msystems.00092-16. https://doi.org/10.1128/msystems.00092-16.

(4) Westerholm, M.; Calusinska, M.; Dolfing, J. Syntrophic Propionate-Oxidizing Bacteria in Methanogenic Systems. *FEMS Microbiol. Rev.* **2022**, *46* (2), fuab057. https://doi.org/10.1093/femsre/fuab057.


# Supplementary figures

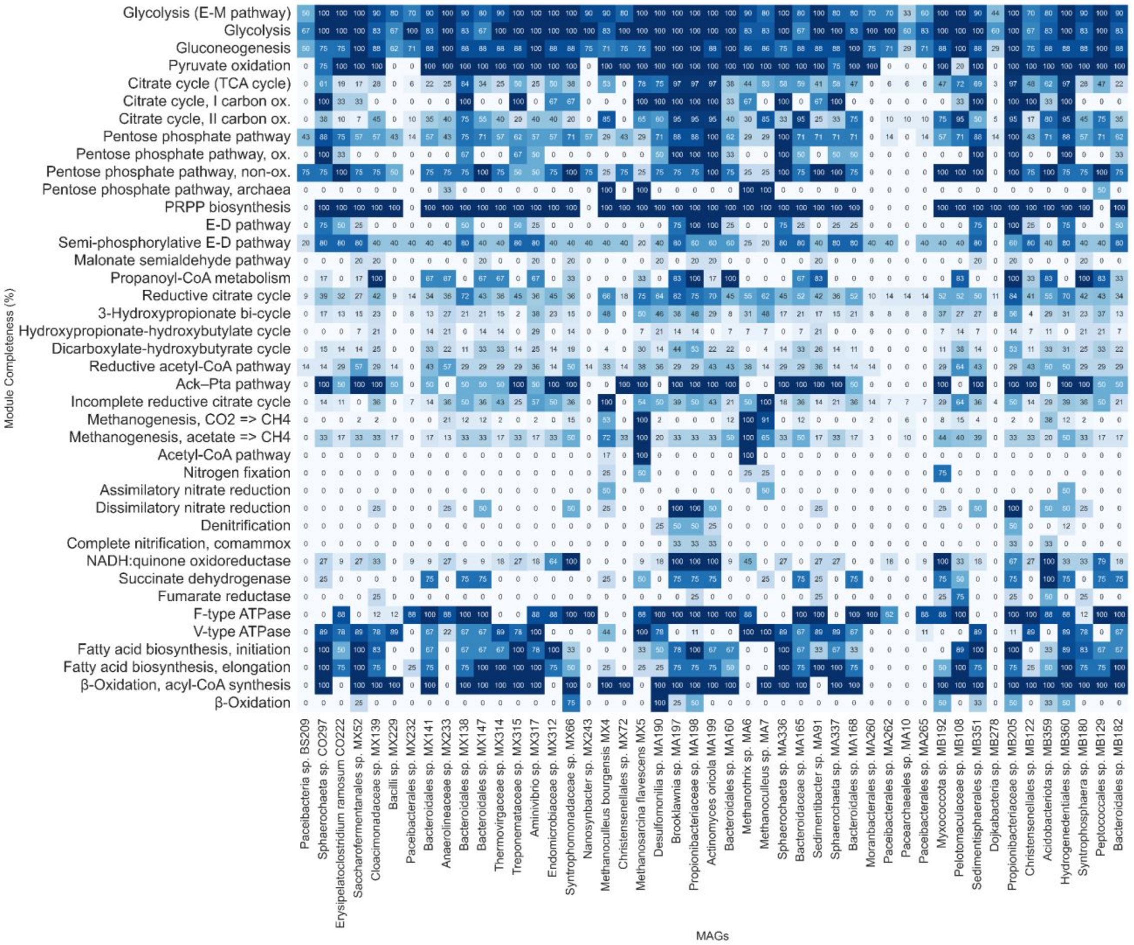


**Figure S1: Module completeness.** Heatmap representing completeness level (reported as a percentage from 0 to 100) of KEGG modules of pathway of interest. Only MAGs with RA > 0.5% were investigated.

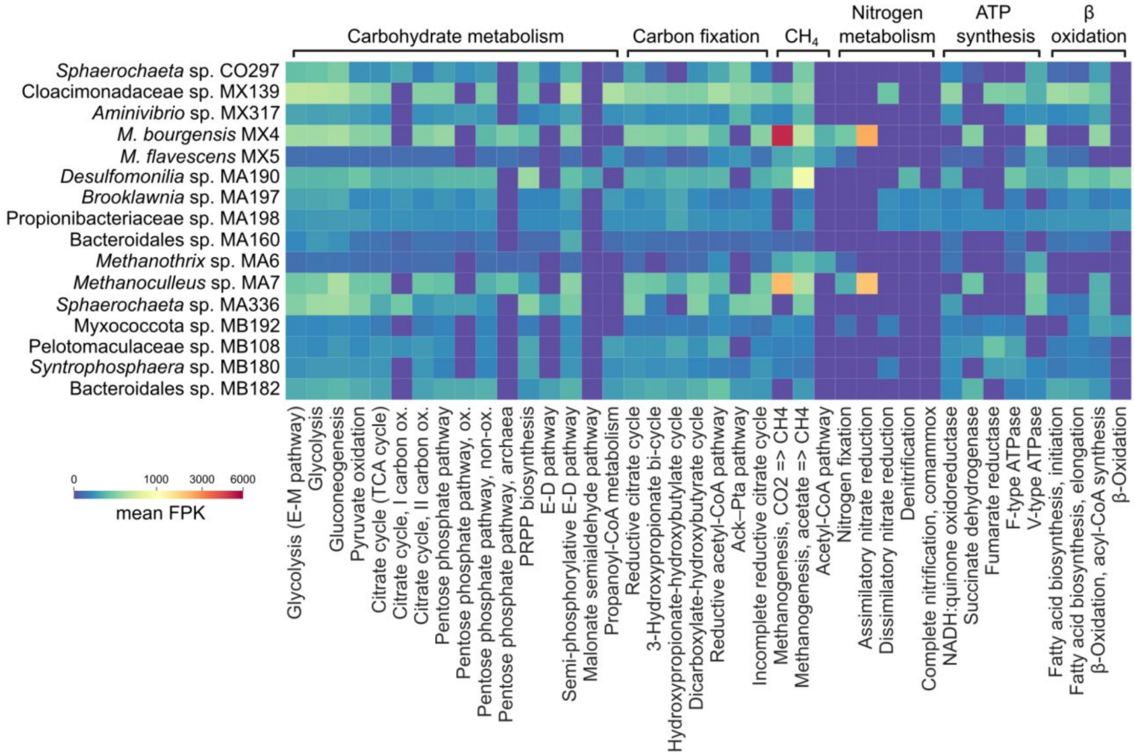


**Figure S2. Metabolic activity of the most abundant MAGs.** The average FPK was calculated for some selected KEGG modules involved in ATP synthesis, carbon fixation, carbohydrate metabolism, beta-oxidation, methanogenesis and nitrogen metabolism. Only MAGs with FPK higher than 50 in one module are displayed. (E-M: Embden-Meyerhof, E-D: Entner-Doudoroff, Ack-Pta:, ox.: oxidative )

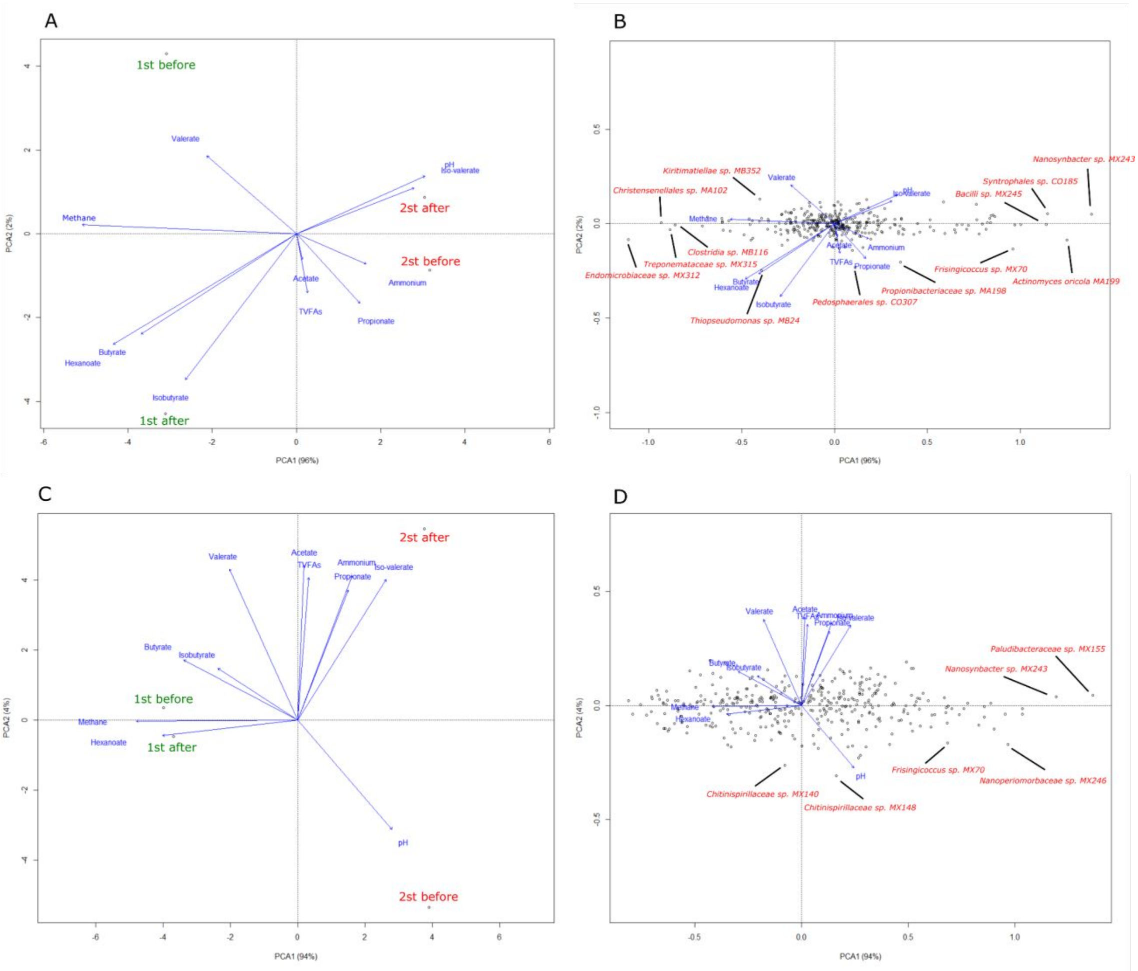


**Figure S3: Physicochemical vs metagenomics and metatranscriptomics genome abundance data.** Principal component analysis (PCA) biplot of metagenomics (A, B) and metatranscriptomics abundance (C, D) and physicochemical concentration separately by samples (A, C) and genomes (B, D). Blue arrows report the direction and strength of the physicochemical parameter. Only the name of outgroup genomes is reported.
